# Efficacy and safety of anlotinib, a multikinase angiogenesis inhibitor, in combination with epirubicin in preclinical models of soft tissue sarcoma

**DOI:** 10.1002/cam4.2941

**Published:** 2020-03-17

**Authors:** Zhi‐Ming Wang, Shi‐Long Zhang, Hua Yang, Rong‐Yuan Zhuang, Xi Guo, Han‐Xing Tong, Yong Zhang, Wei‐Qi Lu, Yu‐Hong Zhou

**Affiliations:** ^1^ Department of Medical Oncology Zhongshan Hospital Fudan University Shanghai China; ^2^ Xiamen Branch Zhongshan Hospital Fudan University Xiamen China; ^3^ Minhang Hospital Fudan University Shanghai China; ^4^ Minhang Hospital Fudan University Institute of Fudan‐Minhang Academic Health System Shanghai China; ^5^ Department of General Surgery Shanghai Public Health Clinical Center Zhongshan Hospital (South Branch) Fudan University Shanghai China; ^6^ Department of General Surgery Zhongshan Hospital Fudan University Shanghai China

**Keywords:** anlotinib, combination, epirubicin, patient‐derived xenografts, soft tissue sarcoma, tyrosine kinase inhibitor

## Abstract

**Background:**

Anlotinib is a novel, orally administered, multitarget receptor tyrosine kinase inhibitor. It functions by inhibiting tumor angiogenesis and proliferative signaling pathways. In this study, we aimed to investigate the efficacy and safety of anlotinib plus epirubicin in a sarcoma patient‐derived xenografts (PDX) model.

**Methods:**

We firstly established a PDX model using fresh tumor tissues that were surgically removed from a patient diagnosed with malignant fibrous histiocytoma. Thirty‐six PDX models were divided into six groups and treated with anlotinib alone (low‐dose, 1.5 or high‐dose, 3.0 mg/kg/day, oral gavage), or with anlotinib plus epirubicin (3.0 mg/kg/once weekly, i.p.) when the tumors grew to 150‐200 mm^3^. After 5 weeks of treatment, the mice were sacrificed, and the tumors were measured by weight and processed for IHC and H&E staining. IHC staining was performed to detect CD31, EGFR, MVD, and Ki‐67 on paraffin sections. H&E stainings were performed to examine the microcosmic changes that occurred in the tumor tissues and myocardium, respectively.

**Results:**

After 5 weeks, treatment with anlotinib or epirubicin alone significantly inhibited tumor growth in the sarcoma PDX model compared with the vehicle control. Tumor volume in the high‐dose anlotinib group was significantly smaller than the low‐dose anlotinib group (*P* < .001). Combined high‐dose anlotinib and epirubicin treatment resulted in the most pronounced tumor inhibition. In the groups treated with the anlotinib‐containing regimen, the expression levels of CD31, EGFR, MVD, and Ki‐67 were significantly low. The weight in each group had no statistical differences; the same applied to the hepatic function, cardiac function, and toxicity.

**Conclusions:**

High‐dose anlotinib combined with epirubicin was an effective and safe therapy for STS.

## Graphical abstract

Soft tissue sarcoma (STS) is a rare and diverse group of solid tumors with poor survival. For the first time, we showed that anlotinib, a multikinase angiogenesis inhibitor, combined with epirubicin was an effective and safe therapy in a sarcoma patient‐derived xenografts model, which suggested combined therapy drug as a promising therapeutic strategy for STS patients.

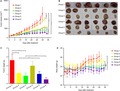

## 1 INTRODUCTION

Soft tissue sarcoma (STS) represents a rare and diverse group of solid tumors that originate from mesenchymal precursors. They possess different clinicopathological features and biological behaviors.^1^ STS accounts for approximately 1% of all new adult malignancies, and contributed to 5150 estimated deaths in 2018.^2^ Sixty different subtypes of STS have been reported^3^; most of them being highly malignant and lack effective medicinal management.

The definitive treatment for localized disease is surgical resection, with or without the combination of radiotherapy for selected STS patients.^4^, ^5^ Systemic treatment with chemotherapy is one of the main therapies for patients with advanced/metastatic disease.^6^ However, there is no standard management for STS patients who progressed after first‐line chemotherapy.^7^, ^8^ The median overall survival for these patients is only 8 to 12 months.^9^ Hence, there is an urgent need to develop new therapies for this condition.

Anlotinib, also known as AL3818, is a novel, orally administered, multitarget tyrosine kinase inhibitor (TKI) that blocks tumor angiogenesis and proliferation. Chi et al reported that anlotinib possesses a satisfactory clinical efficacy, and is well tolerated in advanced STS patients at a daily dose of 12 mg.^10^ Unfortunately, to date, no preclinical studies have been performed to investigate its safety and efficacy in combination with cytotoxic chemotherapy in these patients. Hence, we established a sarcoma PDX model and evaluated the safety and efficacy of anlotinib in combination with epirubicin in this study.

## 2 MATERIALS AND METHODS

### 2.1 Materials

Anlotinib and epirubicin were kindly provided as a gift from Jiangsu Chia‐tai Tianqing Pharmaceutical Co., Ltd (Nanjing, People's Republic of China). Antibodies to EGFR, CD31, MVD, and Ki‐67 were purchased from CST (Beverly, MA, USA).

### 2.2 Establishment of PDX models and treatment protocol

Six‐to‐eight‐week‐old female BALB/c nude mice were purchased from Shanghai Sippr‐BK Laboratory Animal Corporation (Shanghai, China), and housed maintained under specific pathogen‐free (SPF) conditions on a 12/12 hours light/dark schedule with food and water ad libitum. All animal experiments were approved by the Ethic Committee of Zhongshan hospital, and were performed according to the NIH Guide for Care and Use of Laboratory Animals. The PDX mouse models were established using surgically removed fresh tumor tissues from a patient who was histologically diagnosed with malignant fibrous histiocytoma. Tumors sizes were measured using digital calipers every 3 days. Tumor volume (mm^3^) was calculated by the following formula: *V* = 0.5 × (length × width^2^) mm^3^. Xenografts from the fourth generation were used for the experiments, once the tumor volume reached 150‐200 mm^3^ as we previously described.^11^ Mice with fourth generation xenografts were randomized into six groups (6 mice per group): Group 1, vehicle control (DD‐H_2_O, orally gavage, daily); Group 2, low‐dose anlotinib (1.5 mg/kg, oral gavage, daily); Group 3, high‐dose anlotinib (3.0 mg/kg oral gavage, daily); Group 4, epirubicin (2.5 mg/kg, intraperitoneal injection, weekly); Group 5, low‐dose anlotinib (1.5 mg/kg, oral gavage, daily) + epirubicin (2.5 mg/kg, intraperitoneal injection, weekly); Group 6, high‐dose anlotinib (3.0 mg/kg oral gavage, daily) + epirubicin (2.5 mg/kg, intraperitoneal injection, weekly). Anlotinib was suspended in DD‐H_2_O and was thoroughly mixed to obtain a 0.3 mg/mL solution. Epirubicin was diluted with 0.9% Nacl to produce a 0.25 mg/mL solution. Each group was treated for 35 days. Mice were killed when their tumor volumes exceeded 2000 mm^3^.

### 2.3 Histological analysis

Fresh xenograft specimens were fixed in 10% formalin overnight at 4°C, and subsequently dehydrated and embedded in paraffin. Then, they were stained with hematoxylin/eosin (H&E) according to standard protocols. Immunohistochemistry (IHC) was performed using the following primary antibodies: EGFR, CD31, MVD, and Ki‐67. Staining procedures were conducted according to the manufacturers' protocols. The expression of EGFR, and MVD were determined by counting integrated optical density (IOD). The percent of microvessel area stained by CD31 in each field were measured and expressed as a vessel area percent. Furthermore, the expression level of Ki‐67 was defined as the percentage of Ki67‐positive cells. The H&E analysis was performed using a BHS System Microscope (Olympus Corporation). All section analyses were blinded.

### 2.4 Statistical analysis

Data are shown as the mean ± SD. Statistical analysis for parametric variables was done with the independent two‐sample t‐test. For nonparametric variables the Mann‐Whitney U‐test was conducted. The SPSS version 22.0 and GraphPad Prism software version 8.0 (GraphPad Software, Inc) were used. The statistically significant *P* values were suggested in the figures as follows: ***P < .001, **P < .01, *P < .05.

## 3 RESULTS

### 3.1 Efficacy of anlotinib and epirubicin in the sarcoma PDX model

**Figure 1:**
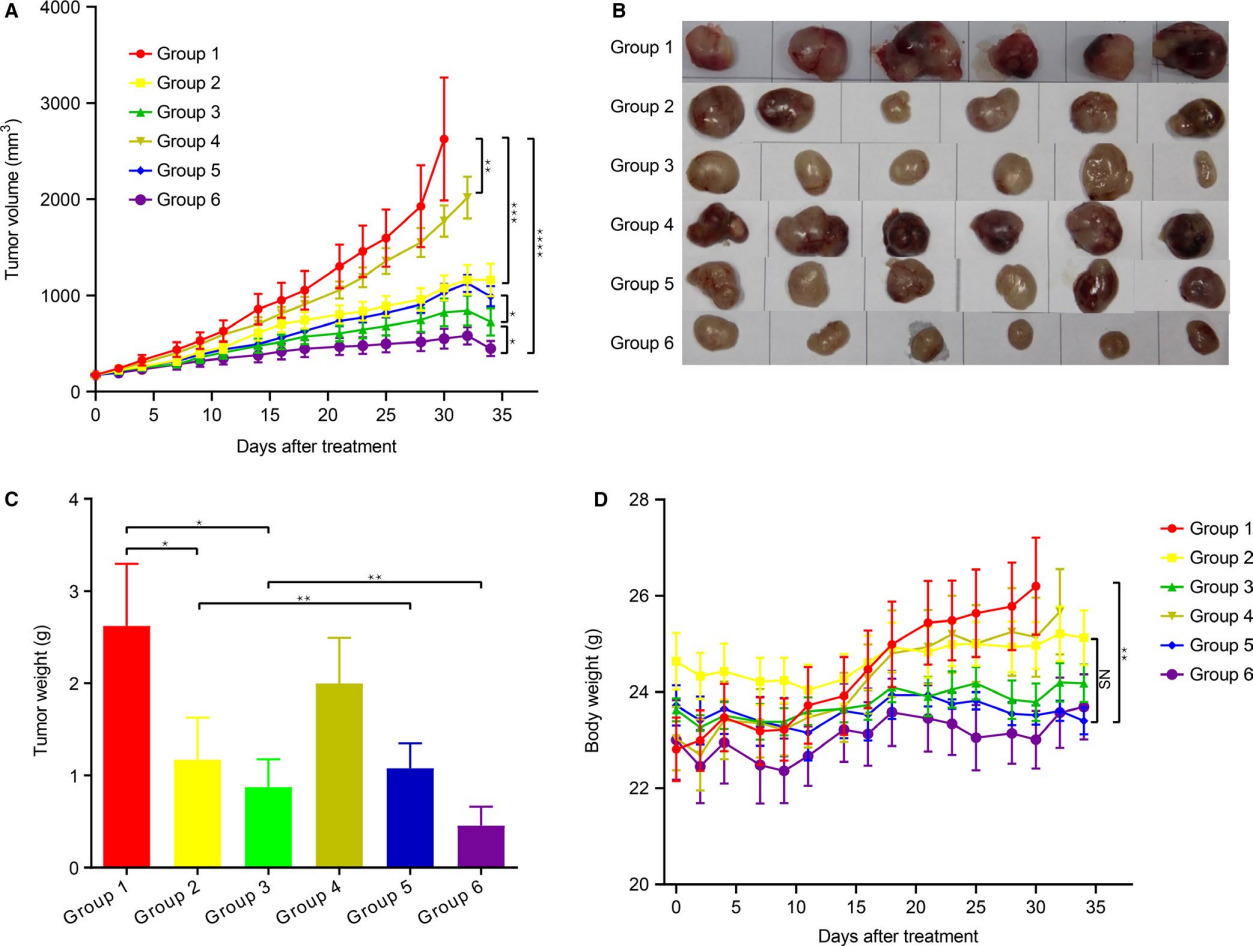
Effect of Anlotinib on the sarcoma PDX model. A, Tumor growth curve of each group. B, Macroscopic examination of the resected tumor. C, Tumor weight of each group. D, Body weight changes over the treatment period.Group 1, vehicle control (DD‐H2O); Group 2, low‐dose anlotinib (1.5 mg/kg per day); Group 3, high‐dose anlotinib (3.0 mg/kg per day); Group 4, epirubicin (2.5 mg/kg per week); Group 5, low‐dose anlotinib (1.5 mg/kg per day) and epirubicin (2.5 mg/kg per week); Group 6, high‐dose anlotinib (3.0 mg/kg per day) and epirubicin (2.5 mg/kg per week)

To explore the efficacy of anlotinib or epirubicin monotherapy in the human sarcoma PDX, we administered epirubicin and anlotinib, respectively. We found that using monotherapy (anlotinib or epirubicin) significantly inhibited tumor growth compared to the vehicle control (anlotinib: *P* < .001; epirubicin: *P* < .001), recorded on Day 35 after treatment. Notably, tumor volume in the high‐dose anlotinib group was significantly smaller than that in the low‐dose anlotinib group (*P* < .05) (Figure 1A), which indicated that anlotinib exerted antitumor activity in a dose‐dependent manner. In addition, epirubicin monotherapy also lead to tumor growth inhibition, but just less pronounced compared to anlotinib.

### 3.2 Efficacy of combined treatment in the sarcoma PDX model

We next evaluated the antitumor activity of anlotinib in combination with epirubicin in the PDX models. Results showed that the combination high‐dose anlotinib and epirubicin showed stronger tumor inhibition than low‐dose anlotinib plus epirubicin, or anlotinib alone (*P* < .001) or epirubicin alone (*P* < .001). This also applied to the vehicle control (*P* < .001) (Figure 1B). At the end of treatment, tumor weights in the combination group were significantly decreased compared to other groups (Figure 1C).

### 3.3 Safety of combined treatment on body weight, hepatic function, and cardiac function in the sarcoma PDX model

**Figure 2:**
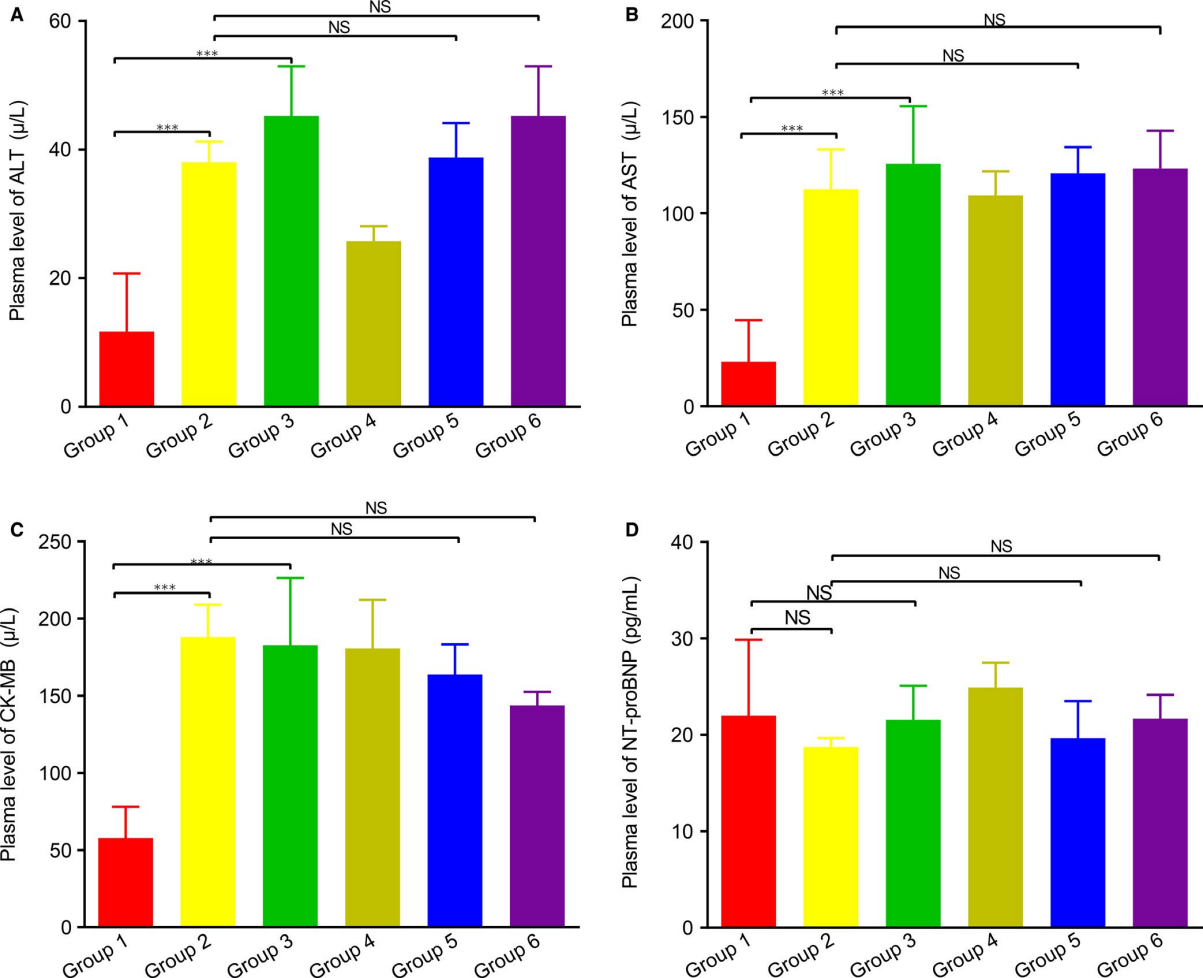
The hepatic and cardiac function of mice at the end of study. A, Serum levels of ALT. B, Serum levels of AST. C, Serum levels of CK‐MB. C, Serum levels of BNP. Group 1, vehicle control (DD‐H2O); Group 2, low‐dose anlotinib (1.5 mg/kg per day); Group 3, high‐dose anlotinib (3.0 mg/kg per day); Group 4, epirubicin (2.5 mg/kg per week); Group 5, low‐dose anlotinib (1.5 mg/kg per day) and epirubicin (2.5 mg/kg per week); Group 6, high‐dose anlotinib (3.0 mg/kg per day) and epirubicin (2.5 mg/kg per week)

Mice body weight was measured before and after treatment. Overall, the combined high/low‐dose anlotinib and epirubicin treatment was well tolerated. No obvious weight loss was observed during the experimental period in any group (Figure 1D). In addition, no significant deterioration was observed to the hepatic function or cardiac function compared to anlotinib monotherapy. The serum alanine aminotransferase (ALT), aspartate aminotransferase (AST), creatine kinase isoenzyme (CK‐MB), B‐type natriuretic peptide (BNP) levels of each group are summarized in Figure 2.

**Figure 3:**
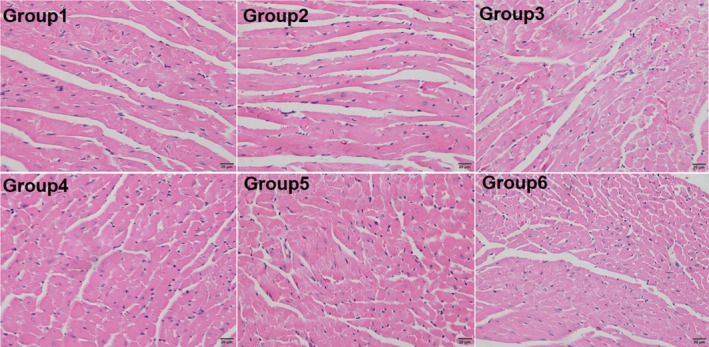
Anlotinib promoted tumor apoptosis and necrosis. The H&E staining of representative tumor sections in each group. ×400. Group 1, vehicle control (DD‐H2O); Group 2, low‐dose anlotinib (1.5 mg/kg per day); Group 3, high‐dose anlotinib (3.0 mg/kg per day); Group 4, epirubicin (2.5 mg/kg per week); Group 5, low‐dose anlotinib (1.5 mg/kg per day) and epirubicin (2.5 mg/kg per week); Group 6, high‐dose anlotinib (3.0 mg/kg per day) and epirubicin (2.5 mg/kg per week)

**Figure 4:**
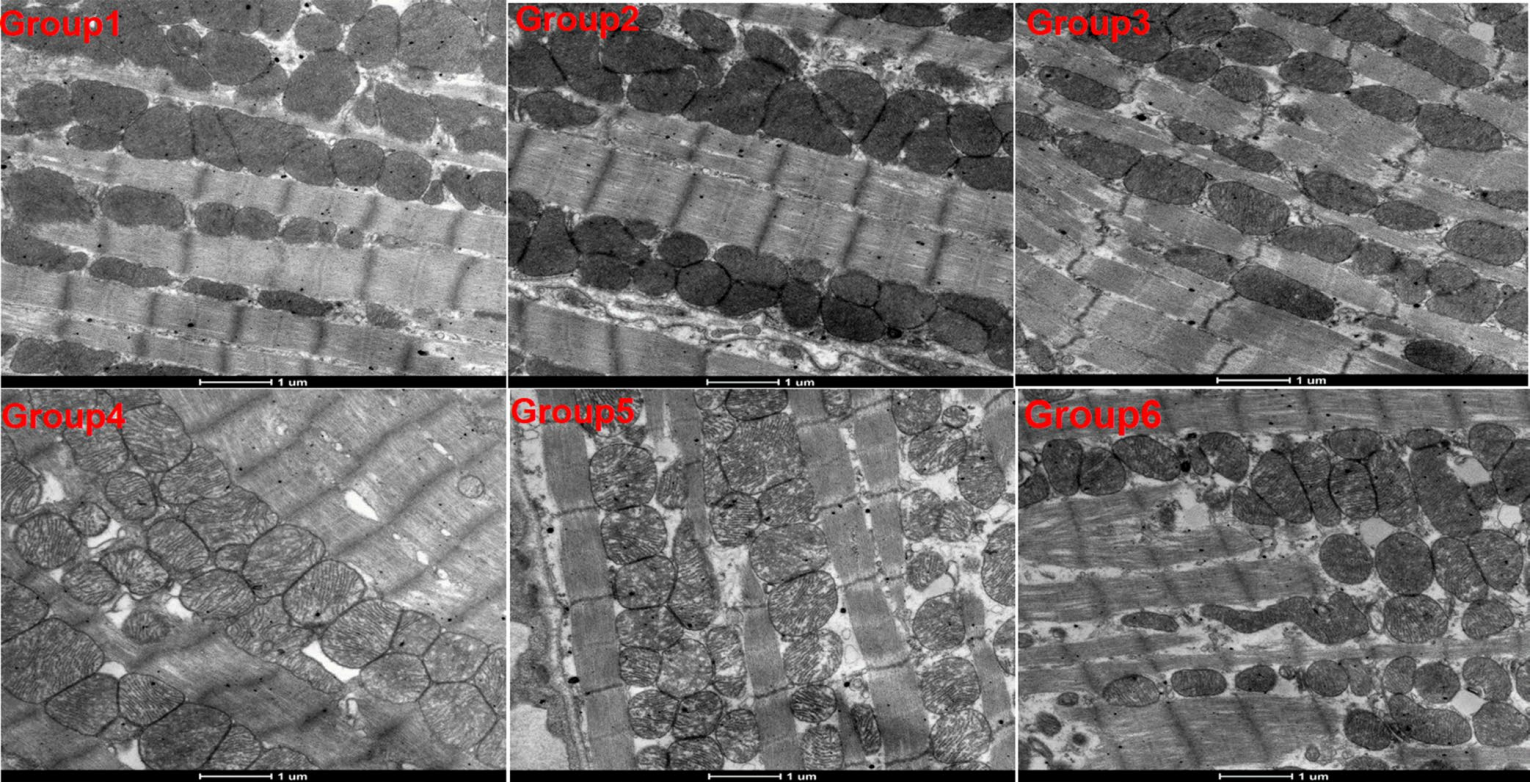
The representative myocardial cell morphology of mice in each group under the H&E staining. ×400. Group 1, vehicle control (DD‐H2O); Group 2, low‐dose anlotinib (1.5 mg/kg per day); Group 3, high‐dose anlotinib (3.0 mg/kg per day); Group 4, epirubicin (2.5 mg/kg per week); Group 5, low‐dose anlotinib (1.5 mg/kg per day) and epirubicin (2.5 mg/kg per week); Group 6, high‐dose anlotinib (3.0 mg/kg per day) and epirubicin (2.5 mg/kg per week)

To determine if the combination of high‐dose anlotinib and epirubicin treatment would aggravate cardiotoxicity in the mice, we performed histological analysis of myocardium. As shown in Figure 3, except for Group 3 (slight vacuolation), no obvious cardiac lesions were found in the remaining groups. Furthermore, the structure of the mitochondria and endoplasmic reticulum was integrated without swelling under electron microscope (Figure 4).

### 3.4 Impact of combination treatment on the histology of the sarcoma PDX model

**Figure 5:**
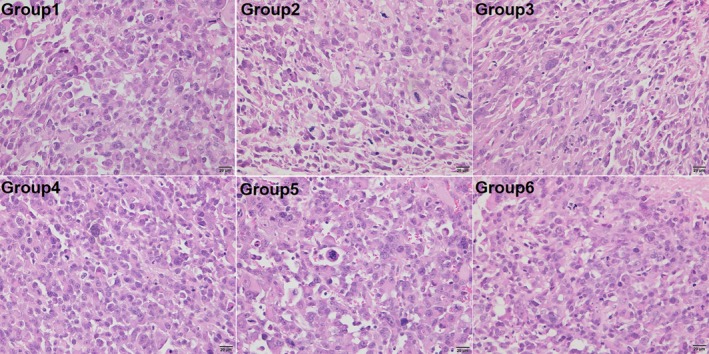
The representative myocardial cell morphology of mice in each group under the H&E staining. ×400. Group 1, vehicle control (DD‐H2O); Group 2, low‐dose anlotinib (1.5 mg/kg per day); Group 3, high‐dose anlotinib (3.0 mg/kg per day); Group 4, epirubicin (2.5 mg/kg per week); Group 5, low‐dose anlotinib (1.5 mg/kg per day) and epirubicin (2.5 mg/kg per week); Group 6, high‐dose anlotinib (3.0 mg/kg per day) and epirubicin (2.5 mg/kg per week)

**Figure 6:**
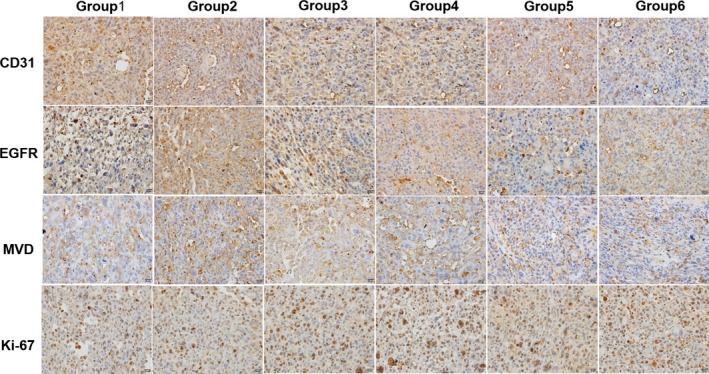
Anlotinib decreased tumor angiogenesis and cell proliferation. IHC analysis of CD31, EGFR, MVD, and Ki‐67 expression of representative tumor sections in each group. ×400. Group 1, vehicle control (DD‐H2O); Group 2, low‐dose anlotinib (1.5 mg/kg per day); Group 3, high‐dose anlotinib (3.0 mg/kg per day); Group 4, epirubicin (2.5 mg/kg per week); Group 5, low‐dose anlotinib (1.5 mg/kg per day) and epirubicin (2.5 mg/kg per week); Group 6, high‐dose anlotinib (3.0 mg/kg per day) and epirubicin (2.5 mg/kg per week)

Histological analysis of the tumors was conducted by H&E and IHC staining. We found that the vehicle control tumor mainly comprised of viable tumor cells with little tumor necrosis. In contrast, tumors treated with epirubicin, or anlotinib contained necrosis as well as apoptosis. Necrosis and apoptosis were most notable in the combined high‐dose anlotinib and epirubicin group (Figure 5). To exam the vascular density within tumor tissues, we examined the expression of CD31, EGFR, and MVD by IHC staining. In accordance with the antitumor activity, these proteins were significantly decreased after the anlotinib‐containing treatment (Figure 6). These results suggested that anlotinib exerted antitumor effect against STS tumors through an antiangiogenic activity.

Furthermore, in addition to reducing tumor angiogenesis, we wanted to investigate whether the combination treatment influenced tumor growth by inhibiting cell proliferation. Hence, we calculated the percentage of Ki67‐positive cells within the tumors; we found that combined treatment produced the largest decreases in Ki‐67‐positive cells (Figure 6), which was in accordance with the smallest tumor volume of this group compared to the other groups.

## 4 DISCUSSION

Chemotherapy remains an essential part of multimodal cancer treatment but causes severe short‐ and long‐term toxicities. Recently, a large systematic review and meta‐analysis involving 5044 patients showed that multiagent chemotherapy merely achieved mild improvements in survival for STS patients.^12^ In the modern era of cancer therapeutics, there is a drive towards combined treatment modalities in chemotherapy, as well as targeted agents that enhance cancer‐specific killing while reducing toxic side effects. Hence, we first established a human sarcoma PDX model to investigate the efficacy and safety of the multitarget TKI anlotinib and combined it with the first‐line chemotherapy agents epirubicin.

At present, many comprehensive genomic analyses have determined specific molecular alterations in STS.^13^ Vascular endothelial growth factor (VEGF)/vascular endothelial growth factor receptors (VEGFRs) is one of the most recognized major mediators of angiogenesis; it plays a vital role in tumor growth, proliferation, and metastasis.^14^, ^15^ Additionally, aberrant stimulation of the fibroblast growth factor (FGF)/fibroblast growth factor receptor (FGFR) axis activates a series of downstream signaling pathways, such as the PI3K/Akt/mTOR, and Raf/Mek/Erk pathways,^16^, ^17^ which thus evokes tumor aggression and resistance to chemotherapy.^18^, ^19^ Apart from the proangiogenic pathway, regulators in the proliferative pathway, such as platelet‐derived growth factor receptor *α*, *β* (PDGFR *α*, *β*) and stem cell factor receptor (c‐Kit), are also highly malignant phenotypes of STS.^20^, ^21^ Collectively, these findings provided a rationale for using proangiogenic and proliferative regulators as promising therapeutic targets for STS.

**Figure 7:**
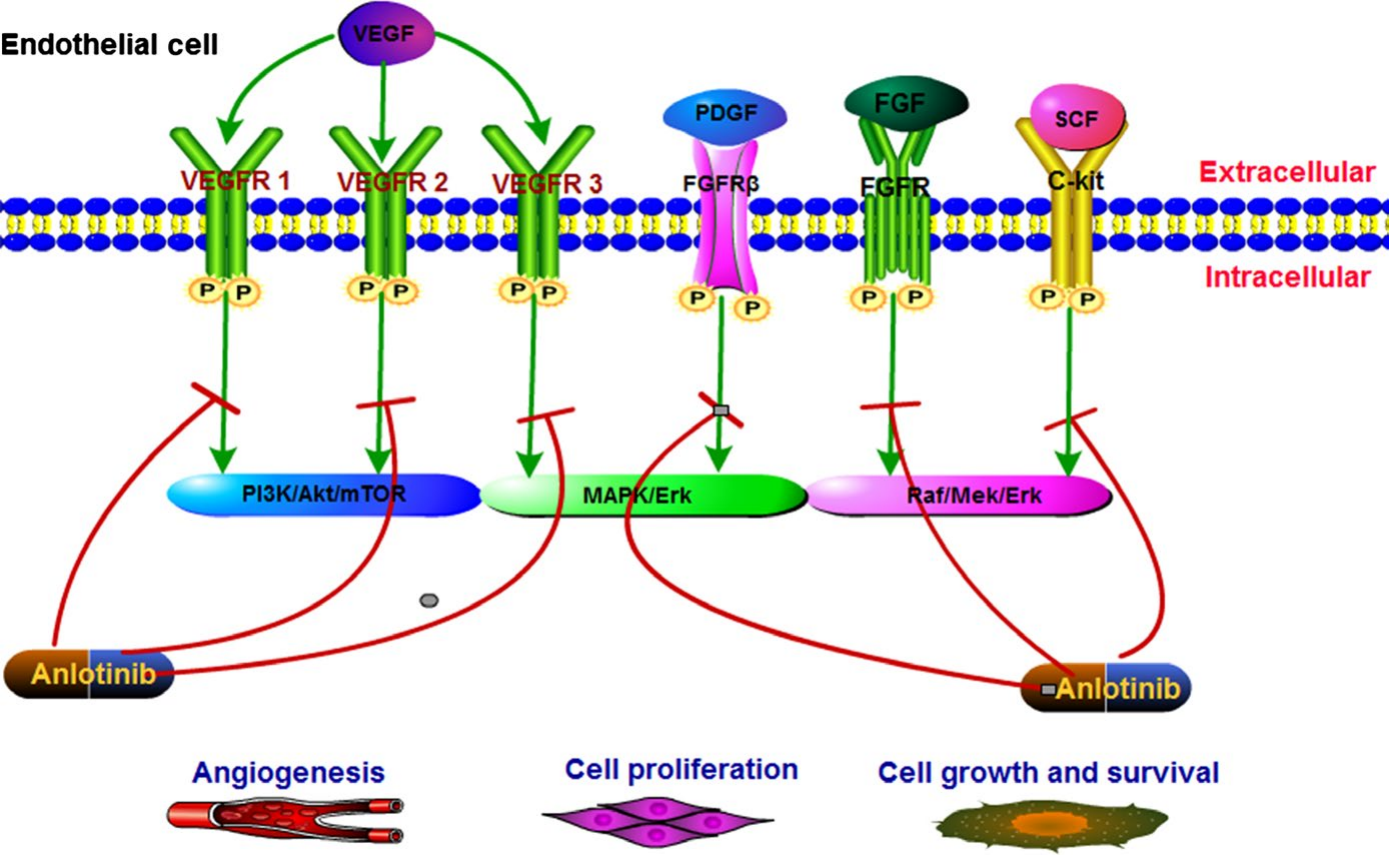
The potential mechanisms of anlotinib in the treatment of STS

Anlotinib is a novel, oral, small‐molecule TKI, that antagonizes multiple tumor proangiogenic and proliferative signaling pathways.^22^ Its prime targets include VEGFR 1 to 3, FGFR 1 to 4, PDGFR *α*, *β*, and c‐Kit (Figure 7). Hence, anlotinib can inhibit more targets and exert a stronger antitumor activity than other TKIs, including sorafenib, sunitinib, and pazopanib.^23^ Preclinical and clinical studies have shown that anlotinib is effective and safe in the treatment of multiple solid tumors.^10^, ^11^, ^24^, ^25^ Currently, there are also several ongoing Phase I/II clinical trials for different types of solid tumors in China and other countries.

In 2018, researchers from China conducted a multicenter, phase II study to explore the role of anlotinib in patients with advanced STS who had progressed after previous conventional treatments.^26^ For the 166 patients enrolled, the progression‐free rate (PFS) at 12 weeks was 57.23%, median PFS was 5.63 months, and the objective response rate was 11.45%. Generally, anlotinib resulted in satisfactory clinical benefits for several subtypes of STS, particularly for synovial sarcomas, leiomyosarcomas, and alveolar soft part sarcomas. The most common adverse events were hypertension (4.8%), triglyceride elevation (3.6%), and pneumothorax (2.4%). All of them were controllable and no treatment‐related death occurred. Recently, a phase IIB study, which included a larger number of patients, further confirmed the efficacy and safety of anlotinib in advanced STS.^27^

In our study, mice were treated daily with anlotinib (1.5 or 3.0 mg/kg), whereas epirubicin (2.5 mg/kg) was administered by intraperitoneal injection once a week for 5 weeks. We found that anlotinib had a strong antitumor effect in a dose‐dependent manner, and a daily dose of 3 mg/kg was sufficient to significantly inhibit tumor growth. When in combination with epirubicin, the antitumor effect of anlotinib was improved. One feature of our study was that it was designed to fully take into account the potential adverse events attributed to the combination of epirubicin. Epirubicin was introduced to improve the safety profile of doxorubicin. It tended to be slightly less efficacious than doxorubicin, but produced less myelotoxicity, as well as reduced nausea and vomiting.^28^, ^29^ However, in a study that included 314 patients with advanced STS, two different schedules of high‐dose epirubicin failed to improve survival when compared to a standard dose of doxorubicin (75 mg/m^2^); moreover, the previous advantage regarding reduced toxicity did not apply.^30^ In our study, the cumulative dose of epirubicin for mice was set to be equal to a human dose of 100 mg/m^2^, which is used in initial, recurrence and metastasis STS in the clinic.^31^, ^32^ We biopsied myocardial tissues for H&E and electron microscopic sections to observe the histopathology and ultrastructure of the myocardium under an optical microscope and transmission electron microscope, respectively. The results showed that combination treatment did not aggravate toxicity in the heart. Furthermore, the combination was well tolerated, as indicated by body weights, hepatic, and cardiac function.

In conclusion, our study indicated that anlotinib had a satisfactory antitumor effect on the sarcoma PDX model in a dose‐dependent manner. The combination of high‐dose anlotinib and epirubicin showed an enhanced effect compared to either drugs being used as monotherapy. The adverse event was slight and acceptable. In light of these promising results, in our next study, we aim to conduct a single‐arm, phase II study to investigate the efficacy and safety of high‐dose anlotinib plus epirubicin as a palliative first‐line treatment for patients with advanced/metastatic STS.

## CONFLICT OF INTEREST

All the authors have no conflicts of interested to declare.

## AUTHOR CONTRIBUTIONS

ZW, SZ, HY, and YZ conducted all experiments and analyzed the data. ZW and SZ provided support with experimental techniques. RZ, XG, HT, YZ, and WL provided clinical samples. YZ and ZW wrote the manuscript. YZ and SZ contributed to manuscript revision. All authors read and approved the final manuscript.

## CONSENT FOR PUBLICATION

All contributing authors agree to the publication of this article.

## Data Availability

The data that support the findings of this study are available on request from the corresponding author. The data are not publicly available due to privacy or ethical restrictions.
